# Differences in social adjustment during adulthood between adoptees with high and low risk for schizophrenia spectrum disorders – the Finnish adoptive family study of schizophrenia

**DOI:** 10.1007/s00406-024-01866-0

**Published:** 2024-07-29

**Authors:** Miia Säkkinen, Helinä Hakko, Karl-Erik Wahlberg, Sami Räsänen

**Affiliations:** 1 Faculty of Medicine, Research Unit of Clinical Medicine, Psychiatry, University of Oulu, Peltolantie 17, PT1, FIN-90220 Oulu, Finland; 2 Department of Psychiatry, Oulu University Hospital, Peltolantie 17, PT1, FIN-90220 Oulu, Finland

**Keywords:** Adoptive family study, Genetic risk, Schizophrenia, Adult adjustment, Social adjustment, Social adaptation

## Abstract

**Objective:**

To investigate differences in social adjustment during adulthood between adoptees with high genetic risk (HR) and low genetic risk (LR) for schizophrenia spectrum disorders.

**Methods:**

This study is a subsample of the Finnish Adoptive Family Study of Schizophrenia. The study sample consisted of 120  adoptees whose biological mothers had DSM-III-R verified schizophrenia spectrum disorders, and 142 socio-demographically matched control adoptees. The social adjustment of the adoptees was assessed using the interview-based Adult Adjustment Scale (AAS).

**Results:**

A lower proportion of the HR adoptees (61.7%) fell into the category of good adaptation compared to LR adoptees (74.6%) (*p* = 0.024). In addition, the median AAS score among HR adoptees was lower compared to LR adoptees (*p* = 0.023). Poorer results among HR adoptees were also found regarding some individual items and the social health -domain within the AAS. The psychiatric morbidity, excluding schizophrenia spectrum disorders, was higher among HR adoptees. Psychiatric morbidity was shown to mediate the association of genetic status to total AAS, and, also to the domain of social health.

**Conclusion:**

According to our results, genetic susceptibility to schizophrenia is associated with weakened social adjustment during adulthood. Although our results demonstrated that psychiatric morbidity has notable effect on the association of genetic status to adult adjustment scores, the impact of other determinants, like psychosocial factors or health-related behaviour, cannot be ruled out. The comparable rearing environment provided by the adoption design in conjunction with reliable diagnostics provide new information on the relation of genetic susceptibility and social adjustment.

## Introduction

Schizophrenia is a psychiatric disorder of partially genetic aetiology [21], with effects extending from emotional and cognitive dysfunctions [4] to effects on behaviour and overall functioning [15], especially social functioning [9]. Closely related to social functioning, impairment can also occur in social adjustment [18]. This concept, also named social adaptation, refers to one’s integration to society, including the ability to live and work with others in harmony and to engage in satisfying interactions and relationships [29]. Among persons with schizophrenia, social adjustment is often severely compromised; they are less likely to live and support themselves independently, and being able to form a stable enough relationship to marry is also rare [13].

Previous studies have revealed evidence suggesting that the weakened social adaptation could extend to concern individuals that are genetically at risk of developing schizophrenia. High-risk individuals have been found to possess schizophrenia-associated traits, such as deficits in cognitive functions [1, 14, 17, 19]. In terms of social adjustment, the most significant findings among high-risk individuals are deficits in social cognitive functions [17], particularly facial emotion recognition [19] and emotional intelligence [1]. In schizophrenia, this kind of impairment in social cognitive functions has been associated with problems in social adjustment, such as poorer social functioning, poorer social skills and poorer independent living skills [6]. With social cognitive deficits occurring in high-risk individuals as well, it can be hypothesized whether corresponding problems in functional outcomes and overall social adjustment could also be recorded amongst them.

The matter of social adjustment in high-risk populations has not been studied comprehensively, although some research is available on specific outcomes of social adjustment, e.g., education, employment, marital status and quality of life [7, 10, 24]. In addition, studies have been conducted on social functioning, an interrelated subject containing various mutual elements with social adjustment. For example, in studies conducted on either social adjustment or social functioning, a mutual finding arising repeatedly has been the display of poorer peer relationships during adolescence among high-risk individuals [8, 11, 12, 28]. Although these findings have applied to adolescents in particular, it is possible that challenges in social adjustment could persist into adulthood.

As for the social adjustment of adult high-risk populations, the results of previous studies have been somewhat inconsistent, reporting poorer outcomes in only some areas of social adjustment, e.g., social functioning [23] and employment rates [24]. In addition, Terzian et al. [24] reported that fewer high-risk males were married compared to males in the general population. It should however be noted that these results could partly be explained by psychiatric morbidity rather than genetic susceptibility, since the studies were conducted on first-degree relatives of persons with schizophrenia without excluding any psychiatric disorders among the subjects. More research is needed for attaining a better understanding of the impact of genetic susceptibility to schizophrenia, as well as for identifying possible unfavourable psychosocial factors that may be subject to preventative measures among high-risk individuals.

The aim of our study is to analyse whether there are differences in social adjustment during adulthood between adopted-away offspring of biological mothers with schizophrenia spectrum disorders (high-risk/HR adoptees) and adoptees without genetic susceptibility to schizophrenia (low-risk/LR adoptees). The adoption design enables studying the association of genetic high risk and social adjustment without the possible confounding effect that maternal schizophrenia may have on the rearing environment. To our knowledge this is the first study to investigate the adult adjustment of HR and LR individuals in a setting where the rearing environment is comparable.

## Material & methods

### Study population

The data used in this study was derived from the nationwide Finnish Adoptive Family Study of Schizophrenia [26]. In the first phase of sampling all women (*n* = 19 447) that had been admitted to psychiatric hospitals between the years of 1960 to 1979 were extracted from Finnish hospital records. Of these women, those who had been diagnosed with schizophrenia or paranoid psychosis and who’s cause of hospitalization had at least once been either of these disorders, were identified. Subsequently, those of these women who had adopted away at least one child, were selected. The diagnoses of the adoptees’ biological mothers were then verified according to the *Diagnostic and Statistical Manual of Mental Disorders*,* Third Edition Revised* (DSM-III-R) criteria [2]. The final diagnostic selection was carried out using the broad definition for schizophrenia spectrum disorders created by Kendler et al. [16], which included the following diagnoses: schizophrenia; schizotypal, schizoid, paranoid, and avoidant personality disorders; schizoaffective, schizophreniform, and delusional disorders; bipolar disorder with psychosis; depressive disorder with psychosis; and psychotic disorder not otherwise specified.

The adopted-away offspring of these women was used as a base for the selection of the HR sample. Of the HR adoptees, those who had been adopted after the age of four, adopted abroad or adopted by a relative, were excluded from the study. After this, the study population included 190 HR adoptees and their adoptive parents, the latter having been included in the study with no diagnostic exclusion criteria. The psychiatric assessment of the adoptive parents was also carried out, but this yielded to no exclusions with the aim of providing a sample of adoptive parents representing the mental health of the general population. In the assessment of the mental health of the adoptive parents it was found that 3.1% of the adoptive mothers and 3.8% of the adoptive fathers had broad schizophrenia spectrum diagnoses, but none of these diagnoses were typical schizophrenia, and in no pair of adoptive parents did both mother and father have schizophrenia spectrum disorders [32].

Matching control families were identified based on the age and sex of adoptees and adoptive parents, the age of adoptees at the time of adoption as well as socioeconomic status (SES) and family structure of the adoptive family. The diagnoses of the LR control adoptees’ (*n* = 192) biological mothers were verified according to the DSM-III-R criteria [2] and those diagnosed with schizophrenia spectrum disorders were excluded. A more thorough description of the study design, selection and exclusion criteria is presented in previous articles about the Finnish Adoptive Family Study [25–27].

**Fig. 1:**
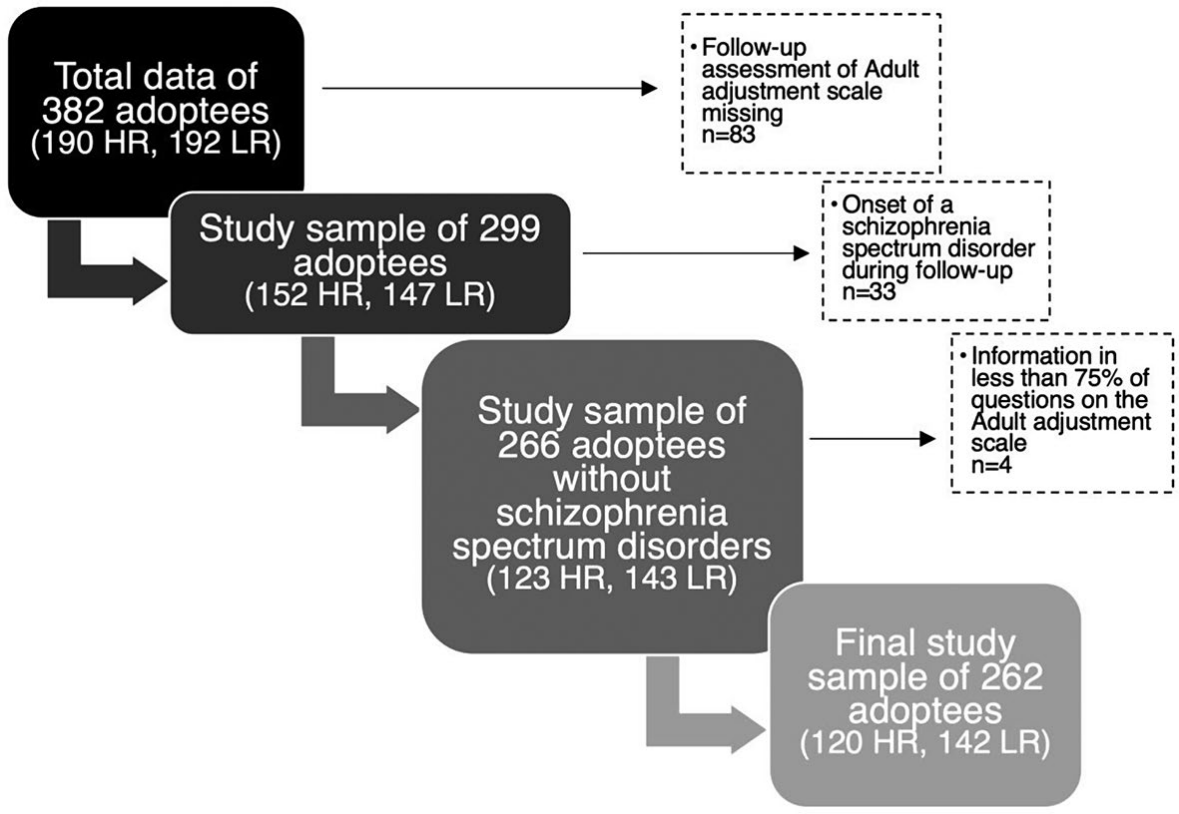
Selection of the study sample consisting of high-risk (HR) and low-risk (LR) adoptees (Figure created with MS Office Word SmartArt-tool)

The selection of adoptees for the present study is presented in Fig. 1. This study utilised the follow-up information collected in adulthood. Adoptees who lacked the follow-up assessment of the Adult Adjustment Scale (AAS), were excluded. In addition, adoptees that were diagnosed with a broad schizophrenia spectrum disorder during follow-up were excluded from statistical analyses, with the aim of avoiding the possible confounding effects of the disorders. Lastly, adoptees with information in less than 75% of the questions on the AAS, were excluded. After these exclusions, the final subsample of this study included 120 HR and 142 LR adoptees.

### Diagnostic procedures & reliability of evaluation

The data on the psychiatric diagnoses of the adoptees and their biological mothers was gathered from hospital records and national registers, and further psychiatric assessment was conducted by interviewing the adoptees, their family members and other informants in initial evaluation as well as during follow-up. This evaluation of adoptees and their biological mothers was carried out by experienced research psychiatrists. The interviewing researchers were blind to the genetic risk statuses of the adoptees. The interrater reliability of the research diagnoses was evaluated during the collection of data [25] with three different procedures to ensure diagnostic reliability. As the first procedure, the kappa coefficient for interrater reliability was tested with a result of 0.80 at initial evaluation and 0.71 at follow-up. Secondly, every 30th subject was re-evaluated by a second rater that performed an independent review and diagnosis, and the kappa coefficient for this action was 0.77. Thirdly, all “problem cases” were re-assessed thoroughly. Two kinds of problem cases were handled: those that displayed disagreement in the random reliability checks, and those in which the rater’s lever of certainty regarding a schizophrenia-related diagnosis was low. The procedure for handling these cases included another rater’s independent assessment, and as required, a case conference for discussing the matter until consensus was reached. The researchers that carried out the follow-up interviews were blinded to the results of all preceding assessments of the adoptees. At the time of the follow-up assessment, the mean age of HR adoptees was 36 years and for LR adoptees it was 37 years.

### Assessment of social adjustment

The follow-up information on the adoptees’ social adjustment was gathered in interviews using a scale adapted from the Adult Adjustment Scale [29] created by Professor G.E. Vaillant. The scale was originally developed for the purpose of the Grant Study, a 75-year longitudinal study that followed graduates of Harvard collecting information about their life after university, including social adjustment. In the original scale, social adjustment was divided into four domains: career, social health, psychological health, and physical health. For the purpose of our study, the scale was modified by simplifying the questions and reframing them into a more universal approach. This was necessary considering that, in the original scale, especially items within the domain of career were framed to specifically suit the Harvard educated study population of the Grant Study. In addition, the domain of physical health was excluded from our version of the Adult Adjustment Scale (AAS), due to physical health not being a core area of social adjustment [30]. In the scale adapted for this study, each domain includes five to seven items that can be affirmed or denied. Each affirmative equals one point, and the maximum score is 19. Based on the answers, the adoptees are then categorized into three social adaptation levels: good adaptation (a score of 14 or more), average adaptation (a score of 8 to 13) and worst adaptation (a score of 7 or less). The adaptation levels and their scores were derived from the original scale by G.E. Vaillant. The interviews that provided information for utilizing the AAS were conducted in the follow-up phase of the study that took place when the mean age of HR adoptees was 36 years and the mean age of LR adoptees was 37 years. Each participant was assessed in one follow-up interview that was conducted by an experienced research psychiatrist. The research psychiatrists that conducted the assessment were blinded to all prior evaluations of the subjects and their biological and adoptive parents.

### Background characteristics

Apart from the genetic susceptibility to schizophrenia, background characteristics of the adoptees included their gender, age at the time of assessing social adjustment, marital status, level of education, SES and psychiatric disorders diagnosed during follow-up. The psychiatric diagnoses were verified by the DSM-III-R [2] criteria, and after excluding the broad schizophrenia spectrum disorders, they were categorized into four diagnostic groups: no psychiatric disorder, depressive & anxiety disorders, personality disorders and other psychiatric disorders.

### Statistical methods

Statistical significance of group differences in continuous variables was assessed by one-way Analysis of Variance (ANOVA) or Mann-Whitney U-test. Statistical significance of group differences in categorical variables was assessed by Pearson Chi-Square test. A two-way ANOVA was performed to explore the effect of psychiatric disorders outside of the schizophrenia spectrum (no psychiatric disorders, depressive/anxiety disorders, personality disorder, other psychiatric disorders) and genetic status (HR, LR) of the adoptees on the total AAS score. A two-way MANOVA was conducted to assess the impact of a psychiatric disorder and genetic status of the adoptees on the scores on the AAS and its domains (career, social health, psychological health), simultaneously. The classical approach to mediation [3] was utilized to analyze, whether psychiatric morbidity (no, yes: mediator, M) mediates the association between genetic status (LR, HR; independent variable, IV) and the AAS score (dependent variable, DV). According to that approach, a so-called complete mediation occurs when IV significantly predicts DV, IV significantly predicts M, and after entering M to the IV-DV relationship, M correlates significantly with DV and the effect of IV to DV reduces to be non-significant. A partial mediation happens when that effect does not reduce to be non-significant, but it is reduced in magnitude. The Sobel test [22] was used determine whether the reduction in the effect of IV to DV, after including M in the model, is significant, and, thus, whether the mediation effect is statistically significant. The statistical software used for the analyses of this study was IBM SPSS Statistics 25. All tests were two-tailed and the limit for statistical significance was set at *p* ≤ 0.05.

## Results

### Background characteristics

Socio-demographic characteristics of the adoptees are presented in Table 1. Statistically significant differences between HR and LR adoptees were found only in the onset of a psychiatric disorder during follow-up; HR adoptees developed more psychiatric disorders outside of the schizophrenia spectrum compared to the LR adoptees (*p* = 0.013).

**Table 1:** Socio-demographic characteristics of the adoptees with high (HR) and low risk (LR) for schizophrenia spectrum disorders

	Total (*n* = 262)	HR (*n* = 120)	LR (*n* = 142)	Group difference
	n(%)	n(%)	n(%)	*p* value
Gender				0.959
Male	124 (47.3)	57 (47.5)	67 (47.2)	
Female	138 (52.7)	63 (52.5)	75 (52.8)	
Age*				0.178
18–29	67 (25.6)	36 (30.0)	31 (21.8)	
30–44	129 (49.2)	52 (43.3)	77 (54.2)	
45 or above	66 (25.2)	32 (26.7)	34 (23.9)	
Marital status				0.181
Single	53 (20.2)	29 (24.2)	24 (16.9)	
Married or living common-law	167 (63.7)	71 (59.2)	96 (67.6)	
Divorced	37 (14.1)	16 (13.3)	21 (14.8)	
Widowed	5 (1.9)	4 (3.3)	1 (0.7)	
Level of education				0.422
Basic education	24 (9.2)	15 (12.5)	9 (6.3)	
Training via working or courses	48 (18.3)	22 (18.3)	26 (18.3)	
Vocational school	84 (32.1)	34 (28.3)	50 (35.2)	
Upper secondary level education or bachelor’s degree	78 (29.8)	35 (29.2)	43 (30.3)	
University	28 (10.7)	14 (11.7)	14 (9.9)	
Socioeconomic status				0.359
Managerial position	31 (11.9)	13 (10.9)	18 (12.7)	
Small-scale entrepreneur, senior clerk etc.	80 (30.7)	32 (26.9)	48 (33.8)	
Skilled worker, junior clerk etc.	119 (45.6)	56 (47.1)	63 (44.4)	
Labourer, assistant etc.	31 (11.9)	18 (15.1)	13 (9.2)	
Psychiatric disorders diagnosed during follow-up **				
No	159 (60.7)	63 (52.5)	96 (67.6)	0.013
Depressive & anxiety disorders	41 (15.6)	23 (19.2)	18 (12.7)	0.150
Personality disorders	48 (18.3)	28 (23.3)	20 (14.1)	0.054
Other psychiatric disorders	14 (5.3)	6 (5.0)	8 (5.6)	0.820

Total number of cases varies due to missing values in some variables

*Age at the time of assessing social adjustment during follow-up

**Only psychiatric diagnoses outside of the schizophrenia spectrum

Table 2 presents the adoptees’ scores on the AAS and its domains in relation to background characteristics of the adoptees. Regarding the total score on the AAS, lowered scores (worsened adult social adjustment) were associated with HR genetic status (*p* = 0.018), single marital status (*p* < 0.001), lower SES (*p* < 0.001) and the presence of a psychiatric disorder (*p* < 0.001). In the domain of career, lowered scores were associated with lower SES (*p* < 0.001) and the presence of a psychiatric disorder (*p* < 0.001). Further, in the domain of social health, lowered scores were related to HR genetic status (*p* = 0.009), younger age (*p* = 0.001), single marital status (*p* < 0.001), lower SES (*p* = 0.019) and the presence of a psychiatric disorder (*p* < 0.001). In the domain of psychological health, lowered scores associated to lower level of education (*p* = 0.021), lower SES (*p* = 0.019) and the presence of a psychiatric disorder (*p* < 0.001).

**Table 2:** Mean (sd) scores on the Adult Adjustment Scale (AAS) and its domains in relation to background characteristics of the adoptees

	Adult Adjustment Scale (AAS), mean (sd)			
	Total score	Domains		
		Career	Social Health	Psychological Health
Genetic status				
High-risk (HR) adoptees	13.87 (3.43)	3.63 (1.22)	4.32 (1.70)	5.93 (1.44)
Low-risk (LR) adoptees	14.82 (3.00)	3.77 (1.21)	4.82 (1.39)	6.23 (1.21)
*Difference between genetic status*,* p-value*	*0.018*	*0.321*	*0.009*	*0.068*
Gender				
Male	14.35 (3.49)	3.77 (1.30)	4.52 (1.58)	6.06 (1.38)
Female	14.41 (3.00)	3.64 (1.13)	4.64 (1.54)	6.12 (1.28)
*Difference between genders*,* p-value*	*0.899*	*0.390*	*0.532*	*0.718*
Age (in years)				
18–29	13.58 (2.78)	3.40 (1.29)	3.99 (1.24)	6.19 (1.21)
30–44	14.67 (3.37)	3.81 (1.23)	4.78 (1.53)	6.07 (1.30)
45 or above	14.64 (3.30)	3.82 (1.05)	4.82 (1.76)	6.00 (1.50)
*Difference between age-groups*,* p-value*	*0.063*	*0.060*	*0.001*	*0.697*
Marital status				
Single	13.11 (3.37)	3.49 (1.32)	3.58 (1.28)	6.04 (1.49)
Married or living common-law	14.99 (3.02)	3.74 (1.23)	5.04 (1.47)	6.22 (1.12)
Divorced	13.46 (3.22)	3.89 (0.97)	4.00 (1.41)	5.57 (1.77)
Widowed	14.20 (4.38)	3.60 (1.14)	4.60 (2.07)	6.00 (1.41)
*Difference between marital status*,* p-value*	*< 0.001*	*0.445*	*< 0.001*	*0.057*
Level of education				
Basic education	13.42 (4.35)	3.17 (1.46)	4.37 (1.88)	5.87 (1.73)
Training via working or courses	13.71 (3.57)	3.56 (1.17)	4.56 (1.67)	5.58 (1.61)
Vocational school	14.43 (2.76)	3.76 (1.04)	4.32 (1.47)	6.35 (0.98)
Upper secondary level education or bachelor’s degree	14.82 (3.16)	3.74 (1.39)	4.87 (1.40)	6.21 (1.67)
University	15.00 (2.85)	4.14 (0.85)	4.82 (1.66)	6.04 (1.55)
*Difference between level of education*,* p-value*	*0.152*	*0.053*	*0. 189*	*0.021*
Socioeconomic status (SES)				
Managerial position	15.68 (2.97)	4.35 (0.84)	5.16 (1.59)	6.16 (1.51)
Small-scale entrepreneur. senior clerk etc.	15.15 (2.93)	4.15 (0.98)	4.78 (1.47)	6.22 (1.20)
Skilled worker. junior clerk etc.	14.10 (3.96)	3.53 (1.13)	4.42 (1.52)	6.15 (1.16)
Labourer. assistant etc.	12.06 (3.23)	2.58 (1.48)	4.10 (1.68)	5.39 (1.82)
*Difference between SES status*,* p-value*	*< 0.001*	*< 0.001*	*0.019*	*0.019*
Psychiatric disorders during follow-up				
No	15.40 (2.29)	3.99 (1.02)	4.90 (1.42)	6.51 (0.75)
Depressive & anxiety disorders	13.56 (3.15)	3.49 (0.95)	4.41 (1.58)	5.66 (1.51)
Personality disorders	12.15 (4.47)	2.96 (1.65)	3.85 (1.71)	5.33 (1.97)
Other psychiatric disorders	12.93 (2.84)	3.71 (1.07)	4.07 (1.54)	5.14 (1.29)
*Difference between diagnostic groups*,* p-value*	*< 0.001*	*< 0.001*	*< 0.001*	*< 0.001*

*Note*: Higher scores indicate better social adjustment. Total number of cases varies due to missing values in some variables

### Social adaptation

**Fig. 2:**
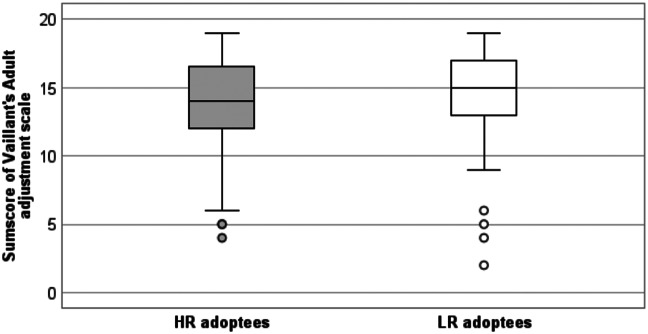
Distribution of total scores on the Adult Adjustment Scale (AAS) among the adoptees with high (HR) and low risk (LR) for schizophrenia spectrum disorders. (Figure created with IBM SPSS Statistics 25)

The distribution of total scores on the AAS among HR and LR adoptees are visualised in Fig. 2. The median score was 15 in the LR group and 14 in the HR group, indicating a statistically significantly (*p* = 0.023) poorer social adjustment in the HR group.

The findings on overall social adjustment according to the social adaptation categories of the AAS are reported in Table 3. A statistically significantly lower proportion of the HR adoptees (61.7%) fell into the category of good adaptation compared to the LR adoptees (74.6%).

**Table 3:** Social adaptation categories of the adoptees with high (HR) and low risk (LR) for schizophrenia spectrum disorders

	Total (*n* = 262)	HR (*n* = 120)	LR (*n* = 142)	Group difference
	n (%)	n (%)	n (%)	*p* value
Social Adaptation category*				
Good adaptation (score 14–19)	180 (68.7)	74 (61.7)	106 (74.6)	0.024
Average adaptation (score 8–13)	70 (26.7)	38 (31.7)	32 (22.5)	0.096
Worst adaptation (score 0–7)	12 (4.6)	8 (6.7)	4 (2.8)	0.138

*Adaptation categories determined by the score on the Adult Adjustment Scale (AAS) adapted from G.E. Vaillant’s original scale

Table 4 shows that in the item-specific analysis within each three domains, statistically significant differences were found in the social health and psychological health -domains of social adjustment. HR adoptees more commonly reported to haveserious problems (scholastic performance, delinquency, psychiatric care) with their own children compared to LR adoptees (*p* = 0.037). HR adoptees were also more likely compared to LR adoptees to have less than an average number of friends (*p* = 0.027). In addition, HR adoptees were more likely to experience chronic feelings of depression or boredom than LR adoptees (*p* = 0.043).

**Table 4:** Responses to individual items of the three domains of the Adult Adjustment Scale (AAS) among adoptees with high (HR) and low risk (LR) for schizophrenia spectrum disorders

	Total (*n* = 262)	HR (*n* = 120)	LR (*n* = 142)	Group difference
	n (%)	n (%)	n (%)	*p*-value
I Career				
Occupational status consistent with educational background or higher	227 (86.6)	101 (84.2)	126 (88.7)	0.279
Financial status consistent with education and/or (family) background	236 (90.8)	107 (89.9)	129 (91.5)	0.662
Occupational success equals or surpasses parents’	197 (75.8)	92 (78.0)	105 (73.9)	0.451
Active participation in extracurricular public service activities	106 (41.2)	46 (39.7)	60 (42.6)	0.639
Satisfaction with career	205 (78.8)	89 (74.8)	116 (82.3)	0.141
II Social health				
Marriage has lasted for 10 years or more	115 (44.2)	49 (41.2)	66 (46.8)	0.362
Not divorced, separated or single	170 (64.9)	72 (60.0)	98 (69.0)	0.128
No serious problems (scholastic performance, delinquency, psychiatric care) with own children	201 (82.0)	84 (76.4)	117 (86.7)	0.037
Contacts with surviving family of origin	212 (81.5)	94 (79.7)	118 (83.1)	0.477
At least average number of friends	245 (94.2)	108 (90.8)	137 (97.2)	0.027
Regularly a member of at least one social club	118 (45.9)	53 (44.9)	65 (46.8)	0.767
Regular pastime or athletic activity that involves others (other than family members)	141 (55.5)	58 (49.6)	83 (60.6)	0.078
III Psychological health				
Ability to spend full allotted vacation time	245 (95.0)	110 (94.0)	135 (95.7)	0.528
Satisfaction with career	207 (79.9)	90 (76.3)	117 (83.0)	0.180
Changes in occupational field in connection with promotion or improvement in personal satisfaction	168 (64.9)	72 (61.0)	96 (68.1)	0.235
No detrimental use of alcohol or psychoactive drugs	238 (91.2)	108 (90.0)	130 (92.2)	0.532
No hospitalization because of mental breakdown, alcohol misuse or somatization	250 (95.8)	113 (94.2)	137 (97.2)	0.230
No chronic feelings of depression or boredom	241 (92.7)	107 (89.2)	134 (95.7)	0.043
No more than 10 visits to psychiatric help	246 (94.6)	111 (92.5)	135 (96.4)	0.162

Total number of cases varies due to missing values in some items

**Fig. 3:**
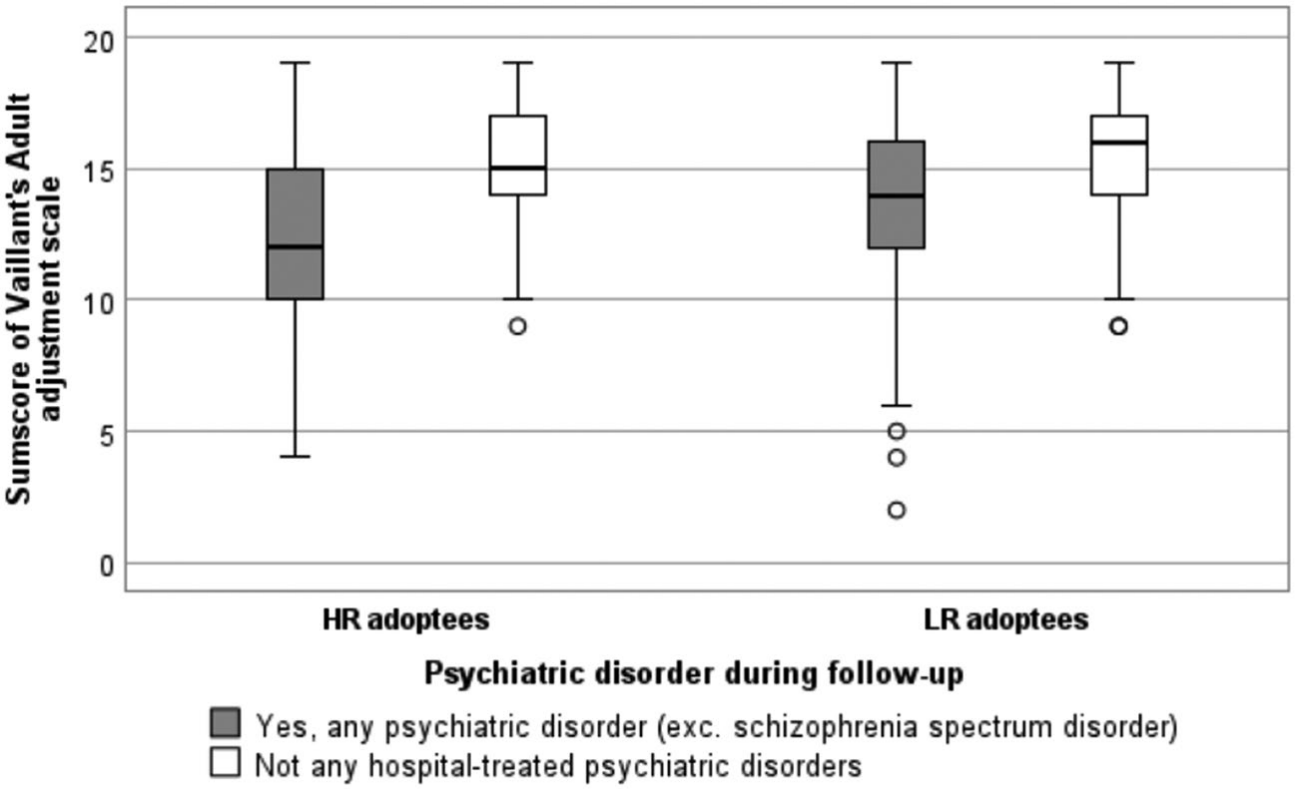
Distribution of total scores on the Adult Adjustment Scale (AAS) among the adoptees with high (HR) and low risk (LR) for schizophrenia spectrum disorders grouped by the presence of a psychiatric disorder. (Figure created with IBM SPSS Statistics 25)

In a further analysis, the association between psychiatric morbidity and the scores on the AAS was observed. As visualised in Fig. 3, we discovered that the adoptees without any psychiatric diagnoses had the best adult adjustment scores compared to those diagnosed with psychiatric disorders outside the schizophrenia spectrum among both HR (*p* < 0.001) and LR (*p* < 0.001) adoptees. However, the median score of HR adoptees was lower even when comparing the scores of HR and LR adoptees in the no psychiatric diagnoses group (15 among HR and 16 among LR adoptees, *p* = 0.527), as well as in the combined psychiatric morbidity group (12 among HR and 14 among LR adoptees, *p* = 0.094), although these results failed to reach statistical significance.

Table 5 reports the mean (sd) scores of the AAS and its domains by psychiatric disorder groups of the HR and LR adoptees. The results of a two-way ANOVA showed a statistically significant effect of psychiatric disorders (*p* < 0.001) and a marginally significant effect of genetic status (*p* = 0.054) on the total AAS score. There was not a statistically significant interaction effect of genetic status and psychiatric disorders on the total AAS score (*F* = 0.720, *p* = 0.541). The results of MANOVA revealed a statistically significant effect of psychiatric disorders (Wilks’ lambda = 0.79, *p* < 0.001), a marginally significant effect of genetic status (Wilks’ lambda = 0.92, *p* = 0.06), but no statistically significant interaction effect (Wilks’ lambda = 0.98, *p* = 0.740) on the career, social health and psychological health domains. The univariate tests showed that psychiatric disorders of the adoptees had a statistically significant effect on all domains of the AAS: career (*F* = 9.81, *p* < 0.001), social health (*F* = 6.07, *p* < 0.001) and psychological health (*F* = 16.42, *p* < 0.001). Genetic status of the adoptees, however, had statistically significant effect only on the social health domain of the AAS (*F* = 5.31, *p* = 0.022), but not on the domains of career (*F* = 0.10, *p* = 0.757) and psychological heath (*F* = 2.46, *p* = 0.118). Further, the interaction effect of psychiatric disorder and genetic status of the adoptees was not significant in any of the domains of the AAS: career (*F* = 0.05, *p* = 0.984), social health (*F* = 0.52, *p* = 0.668) or psychological health (*F* = 1.40, *p* = 0.244).

**Table 5:** Association between psychiatric disorders and mean (sd) scores of the Adult Adjustment Scale (AAS) and its domains among the adoptees with high (HR) and low risk (LR) for schizophrenia spectrum disorders

Psychiatric disorders at follow-up	Adult Adjustment Scale (AAS), mean (sd)			
	Total score	Domains of the AAS		
		Career	Social Health	Psychological Health
HR adoptees (*n* = 120):				
No	15.25 (2.35)	4.00 (0.95)	4.73 (1.56)	6.52 (0.69)
Depressive & anxiety disorders	12.91 (3.53)	3.43 (0.79)	4.17 (1.83)	5.30 (1.66)
Personality disorders	11.96 (4.21)	2.93 (1.68)	3.71 (1.76)	5.32 (1.93)
Other psychiatric disorders	12.83 (2.84)	3.67 (1.37)	3.33 (1.37)	4.83 (1.17)
*Difference between diagnostic groups*,* p-value*	*< 0.001*	*< 0.001*	*0.002*	*< 0.001*
LR adoptees (*n* = 142):				
No	15.49 (2.26)	3.98 (1.07)	5.01 (1.32)	6.50 (0.78)
Depressive & anxiety disorders	14.39 (2.45)	3.56 (1.15)	4.72 (1.18)	6.11 (1.18)
Personality disorders	12.40 (4.91)	3.00 (1.65)	4.05 (1.67)	5.35 (2.08)
Other psychiatric disorders	13.75 (2.71)	3.75 (0.89)	4.62 (1.51)	5.37 (1.41)
*Difference between diagnostic groups*,* p-value*	*< 0.001*	*0.007*	*0.041*	*< 0.001*

*Note*: Higher scores indicate better social adjustment

Table 6 shows the results of a mediation analyses analysing the impact of psychiatric morbidity to the association between the genetic status and the AAS score of the adoptees. The association between genetic status and the total AAS was reduced from b=-0.95 (*p* = 0.018) to b=-0.57 (*p* = 0.125), suggesting so-called complete mediation impact of psychiatric morbidity, and the result was also indicative regarding the psychological health domain (change from b=-0.30, *p* = 0.068 to b=-0.14, *p* = 0.356). A partial mediation was present in social health domain (change from b=-0.50, *p* = 0.009 to b=-0.39, *p* = 0.040). In career domain, although not fulfilling basic assumptions of mediation analysis, the psychiatric morbidity reduced the effect of genetic status to AAS score from b=-0.15 (*p* = 0.321) to b=-0.04 (*p* = 0.771).

**Table 6:** The results of mediation analysis [3] exploring the impact of psychiatric morbidity (mediator) to the association of genetic status (independent variable) with the AAS score (dependent variable) for total scale and its domains (career, social health, and psychological health)

		b	95% CI of b	*p*-value
Total AAS				
Step 1	Genetic status -> AAS score (**direct effect**)	-0.95	-1.73 to -0.17	0.018
Step 2	Genetic status -> Psychiatric morbidity	0.151	0.03 to 0.24	0.013
Step 3	Genetic status -> AAS score (**indirect effect**)	-0.57	-1.31 to 0.16	0.125
	Psychiatric morbidity -> AAS score	-2.49	-3.24 to -1.74	< 0.001
*Sobel test for significance of mediation*				*0.019*
Career domain				
Step 1	Genetic status -> AAS score (**direct effect**)	-0.15	-0.45 to 0.15	0.321
Step 2	Genetic status -> Psychiatric morbidity	0.15	0.03 to 0.27	0.013
Step 3	Genetic status -> AAS score (**indirect effect**)	-0.04	-0.33 to 0.25	0.771
	Psychiatric morbidity -> AAS score	-0.82	-1.00 to -0.42	< 0.001
*Sobel test for significance of mediation*				*0.026*
Social health domain				
Step 1	Genetic status -> AAS score (**direct effect**)	-0.50	-0.88 to -0.12	0.009
Step 2	Genetic status -> Psychiatric morbidity	0.15	0.03 to 0.27	0.013
Step 3	Genetic status -> AAS score (**indirect effect**)	-0.39	-0.76 to -0.02	0.040
	Psychiatric morbidity -> AAS score	-0.73	-1.11 to -0.35	< 0.001
*Sobel test for significance of mediation*				*0.036*
Psychological health domain:				
Step 1	Genetic status -> AAS score (**direct effect**)	-0.30	-0.62 to 0.02	0.068
Step 2	Genetic status -> Psychiatric morbidity	0.15	0.03 to 0.27	0.013
Step 3	Genetic status -> AAS score (**indirect effect**)	-0.14	-0.44 to 0.16	0.356
	Psychiatric morbidity -> AAS score	-1.05	-1.36 to -0.74	< 0.001
*Sobel test for significance of mediation*				*0.018*

## Discussion

Difficulties in social adjustment are an inherent part of schizophrenia and to some extent they have also been recorded in individuals at high genetic risk for schizophrenia. However, regarding the latter, few studies have been able to control the potential environmental effect caused by a first-degree relative living with a psychotic disorder. Thus, the relation of environmental and genetic factors to the differences noted has remained unclear. Information is needed for attaining a better understanding of the potential liabilities caused by genetic susceptibility to schizophrenia, and finding focuses for the development and channelling of preventative measures among high-risk individuals. The adoption design used in this study allowed us to control the rearing environment in relation to genetic susceptibility. By this it was possible to compare structurally measured differences in social adjustment during adulthood between adoptees with and without genetic predisposition to schizophrenia.

As our main finding, we recorded that the social adjustment of high-risk (HR) adoptees was statistically significantly poorer compared to low-risk (LR) adoptees, measured with the interview-based Adult Adjustment Scale (AAS). In addition, only two thirds of HR adoptees were classified as having good social adaptation compared to LR adoptees, from which three quarters had good social adaptation. The three adaptation categories ranging from worst to good were defined according to the original scale created by G. Vaillant, and the adaptation category was determined based on the total score on the AAS. The adoptees who developed schizophrenia spectrum disorders during follow-up were excluded from the analyses to avoid this confounding the results on social adjustment.

In addition to HR genetic status, other background variables that were associated with lowered AAS scores were single marital status, lower socioeconomic status (SES) and the presence of a psychiatric disorder outside of the schizophrenia spectrum. As expected, based on the results of previous studies within the Finnish Adoptive Family Study of Schizophrenia [25], the psychiatric morbidity of HR adoptees was higher in comparison with LR adoptees also outside the schizophrenia spectrum. To elucidate the effect of psychiatric disorders on the poorer social adjustment recorded among HR adoptees, a mediation analysis was performed. The results revealed that psychiatric morbidity holds a notable mediation impact on the poorer social adaptation recorded among HR adoptees. This is plausible considering that even though the adoptees with schizophrenia spectrum disorders were excluded from our study sample, the prevalence of personality disorders was particularly notable among HR adoptees. It should be noted that all psychiatric disorders, personality disorders in particular, have an undisputed impact on social adjustment [20].

Our findings are in line with previous studies conducted without adoption design [23, 24], that have reported poorer performance in different areas of social adjustment among first-degree relatives of persons with schizophrenia. Our results are also in line with a previous study conducted within the Finnish Adoptive Family Study of Schizophrenia by Tikkanen et al. [28], which reported significant deficits among HR adoptees in overall social functioning, in the peer relationships, and involvement in activities during adolescence. The results of our study substantiate previous findings of social adjustment being weakened among persons at high genetic risk for schizophrenia and also support the idea of genetic susceptibility to schizophrenia being linked to poorer social adjustment over the course of life.

Regarding the domain of social health, HR adoptees presented poorer results. Further, impact of psychiatric morbidity on the association between genetic status and social health domain scores was demonstrated. In addition, statistically significantly poorer results were recorded in individual items of the AAS regarding social health: HR adoptees were more likely to have less than an average number of friends, and they were also recorded to have more serious problems with their own children compared to LR adoptees. Regarding the latter, it should be noted that these problems could also be related to characteristics of the offspring of HR adoptees, especially considering that they also possess an increased genetic risk for schizophrenia via their biological grandmothers. In addition to HR genetic status and psychiatric morbidity, the role of socio-demographic factors, like younger age, single marital status, and lower SES of adoptees in our study, may also have had impact on the lowered scores on the social health -domain of the AAS.

Reflecting to previous studies conducted on social adjustment, many of them have also revealed deficits in the social aspect of adjustment in particular. Terzian et al. [24] studied the social adjustment of offspring of persons with schizophrenia, recording poorer rates in marriages of male offspring, although no statistical differences were recorded regarding female offspring. Tamminga et al. [23] compared the social functioning of persons with schizophrenia, their first-degree relatives, and healthy controls, discovering that the group of first-degree relatives performed slightly worse in comparison with the control group and superiorly as compared to the proband group. However, a study by Gkintoni et al. [10] reported no significant differences in the general quality of life or social adaptation, evaluated by the Social Adaptation Self-evaluation Scale (SASS) [5]. The varying results demonstrate that the matter has not been studied widely enough, and the methods of standardization and controlling of numerous possible confounding factors are yet to evolve.

As for the findings regarding the domain of psychological health, the mean score on the AAS of HR adoptees seemed to be lower compared to LR adoptees, although only marginally significantly. In contrast, when analysing the association of different background variables and lowered scores on the psychological health domain of the AAS, other factors, such as psychiatric morbidity as mediation factor, lower SES, and lower level of education, arose to have notable impact on these results. When evaluating the specific items within the psychological domain of adaptation, a statistically significant difference was found in one out of seven items, revealing that HR adoptees were more likely to experience chronic feelings of depression or boredom. The minor findings in the psychological health domain of adaptation could indicate that the strain that genetic susceptibility poses on social aspects of adaptation does not affect psychological adjustment in a way that would result in significant impairment.

Regarding the career-domain of the AAS, no statistically significant differences were recorded between HR and LR adoptees concerning either the mean score of the domain or the individual items within the domain. Further, the impact of psychiatric morbidity as mediation factor on the association of genetic status to the score of the career domain of the adoptees was not found in our study. Reflecting to previous studies concerning the career of HR populations, our result differs from the findings of Terzian et al. [24], who studied the social adjustment of offspring of persons with schizophrenia, recording poorer rates in employment compared to control populations. However, their results could be at least partly related to schizophrenia spectrum disorders rather than genetic susceptibility, since the offspring group did not go through any diagnostic selection. In addition, our results regarding the career of HR individuals can be considered to be in line with evidence recorded regarding education: in a meta-analysis by de Zwarte et al. [7] it was concluded that the relatives of persons with schizophrenia presented similar educational attainment compared to controls. No statistically significant differences emerging in the career domain of the AAS in our study could indicate that the deficits in social functioning linked to genetic susceptibility are delicate enough not to impair occupational success to the extent of statistical significance.

### Strengths & limitations

The data used in this study can be considered to be exceptionally comprehensive, having been derived from the nationwide Finnish Adoptive Family Study of Schizophrenia. To our knowledge, this is also the first study to use adoption design to investigate social adjustment in adulthood. The adoption design provides a unique way to control the effect of rearing environment in relation to genetic susceptibility by eliminating the possible confounding effect that maternal schizophrenia may have on the child.

Another major strength of our study was the data collection procedure including personal interviews and reliable diagnostics that were carried out using structured methods and accomplished by trained researchers. Furthermore, the collection of information on social adjustment during adulthood was implemented by using a valid research instrument, the AAS. The scale was selected based on its availability at the time of initiating the evaluation of the adoptees. Although the approaches to measuring social adjustment are various and no single scale – this one included – has become the standard, a major strength regarding the AAS is that it is not a self-report scale, but a scale that was filled in by trained researchers via interviews. This is often not the case in other scales popularly used for assessing social adjustment today, such as the Social Adjustment Scale – Self-Report (SAS-SR) [31] and the Social Adaptation Self-evaluation Scale (SASS) [5].

At the time of the follow-up assessment, the mean age of HR adoptees was 36 years, and for LR adoptees it was 37 years. Thus, it is not possible to entirely exclude potential onsets of schizophrenia spectrum disorders after the end of follow-up. Hence it is possible that some differences could also be due to premorbid symptoms rather than genetic predisposition only. However, it seems unlikely for these hypothetical cases to possess any potential to confound the results.

Since our study includes many statistical significance tests, the likelihood for change findings (Type I error) cannot be excluded. Also due to small number of cases in subgroup comparison, the lack of statistical power in analyses (Type II error) is also possible. It should also be noted that an even more thorough consideration of environmental factors could further clarify the results – although the adoption design serves as a particular controlling factor, there are still other environmental factors that could contribute to the results. Thereby, in future research, acknowledging factors such as family functioning could provide additional support to the results.

### Conclusion

The results of our study provide further evidence on the potentially endangered adult adjustment of persons that are genetically vulnerable for schizophrenia spectrum disorders. Our results also help to clarify the role of genetic susceptibility by controlling the rearing environment via adoption design. Based on our results, the overall adult social adjustment of high-risk individuals seems to be poorer in comparison with low-risk individuals, with the accentuation of poorer results in the domain of social health. Although psychiatric morbidity was shown to have a notable role in the association of genetic risk status for schizophrenia spectrum disorders to the adult adjustment of the adoptees, the impact of other determinants, like psychosocial factors or health-related behaviour, to this association cannot be ruled out.
